# From Plate to Palette: Dietary Patterns and Their Role in Mucosal Lesions Among North Indian Communities: A cross-sectional study

**DOI:** 10.21142/2523-2754-1204-2024-217

**Published:** 2024-11-23

**Authors:** Kavisha Kapoor Lal, Dhruvendra Lal

**Affiliations:** 1 MDS, Consultant, Department of Dentistry, Sohana Hospital, Sector 77, Mohali, Punjab. India. kavishakapoor49@gmail.com MDS, Consultant Department of Dentistry Sohana Hospital Mohali, Punjab India kavishakapoor49@gmail.com; 2 MD, Assistant Professor, Department of Community Medicine, Dr B R Ambedkar State Institute of Medical Sciences (AIMS) Mohali, Punjab. India. drdhruvlal@gmail.com MD, Assistant Professor, Department of Community Medicine Dr B R Ambedkar State Institute of Medical Sciences (AIMS) Mohali Punjab India drdhruvlal@gmail.com

**Keywords:** Oral lesions, Oral Mucosal Lesions (OML), oral hygiene, oral ulcerations, diet, dietary habits, sweets, tobacco, soft drinks, lesiones orales, lesiones de la mucosa oral (OML), higiene oral, ulceraciones orales, dieta, hábitos dietéticos, dulces, tabaco, refrescos

## Abstract

**Introduction:**

The global oral health scenario, as per WHO 2022 report, states that 3.5 billion people are affected by oral diseases. Despite oral mucous membrane's susceptibility to various diseases, scant information exists on oral lesions. This study aims to assess the impact of dietary habits on oral mucosal lesions in the North Indian population.

**Material and Methods:**

A community-based cross-sectional survey was conducted in a rural area, involving 405 participants. Oral health questionnaires and clinical examinations were utilized for data collection. Chi-square test and Multivariate regression model were used for analysis.

**Results:**

Lesser fruit intake was associated with high prevalence of candidiasis (30%). Sweets consumption was linked with ulcerations (44.4%) an abscess (44.4%) and 4 to 9 times increased risk of oral lesions whereas soft drinks were linked with leukoplakia and candidiasis in 30.8% participants. Tea/coffee consumption was linked to malignant lesions.

**Conclusion:**

Significant proportion had oral mucosal lesions, notably influenced by diet and habits. Tea/coffee intake linked to malignant lesions; sweets to ulcers; soft drinks to leukoplakia. Tobacco showed significant associations. Oral lesion distribution varied across oral cavity regions, emphasizing diverse etiologies.

## RESUMEN

**Introducción:**

El escenario mundial de la salud bucal, según el informe de la OMS 2022, establece que 3500 millones de personas se ven afectadas por enfermedades bucales. A pesar de la susceptibilidad de la mucosa oral a diversas enfermedades, existe escasa información sobre las lesiones bucales. Este estudio tiene como objetivo evaluar el impacto de los hábitos dietéticos sobre las lesiones de la mucosa oral en la población del norte de la India.

**Material y métodos:**

Se realizó una encuesta transversal comunitaria en un área rural, con 405 participantes. Para la recopilación de datos, se utilizaron cuestionarios de salud bucal y exámenes clínicos. Para el análisis, se utilizaron la prueba de chi-cuadrado y el modelo de regresión multivariado.

**Resultados:**

La menor ingesta de frutas se asoció con una alta prevalencia de candidiasis (30%). El consumo de dulces se relacionó con ulceraciones (44,4%), abscesos (44,4%) y un riesgo de 4 a 9 veces mayor de lesiones orales, mientras que los refrescos se relacionaron con leucoplasia y candidiasis en el 30,8% de los participantes. El consumo de té y café se relacionó con lesiones malignas.

**Conclusiones:**

Una proporción significativa presentó lesiones de la mucosa oral, notablemente influenciadas por la dieta y los hábitos. La ingesta de té/café se relaciona con lesiones malignas; los dulces, con úlceras; y los refrescos, con leucoplasia. El tabaco también mostró asociaciones significativas. La distribución de las lesiones orales varió entre las regiones de la cavidad bucal, enfatizando diversas etiologías.

## INTRODUCTION

The WHO defines oral health as the condition of the mouth, teeth, and related structures, crucial for basic functions like eating, breathing, and speaking, and impacting psychosocial aspects like confidence and well-being.^1^ The 2022 WHO report highlights a global oral health crisis, with 3.5 billion people, primarily in middle-income nations, suffering from oral diseases.^2^

Oral mucous membrane is the most susceptible to many diseases and is considered as mirror to oral and general health, but very scarce information is available on oral mucous abnormalities.^3^ Most of the oral health condition are preventable and treatable, provided they are diagnosed at their early stages. Several risk factors can be responsible for these lesions, which may include complex bacterial and viral, metabolic or immunologic alteration due to systemic diseases, drug reactions, or even due to deleterious lifestyle and dietary habits.^3^^,^^4^ Few common oral lesions encountered may include leukoplakia, tori, lichen planus, inflammatory lesions, fibromas, Fordyce's granules, haemangiomas, ulcers, papilloma’s, epuli and varicosities.^5^ Oral leukoplakia, oral submucous fibrosis, and oral erythroplakia have a very high malignant transformation rate and oral lichen planus is one of the potentially malignant disorders in the oral cavity.^6^

Very few studies have been published regarding the influence of dietary habits on various oral lesions and dental caries. There have been a few studies which only reflect tobacco or alcohol as the only risk factors for these lesions.^7^^,^^8^ The present study aims to assess the risk of dietary habits on acquiring various oral mucosal lesions in north Indian population. 

## MATERIALS AND METHODS

The present study was a community based observational cross-sectional survey which was conducted in rural practice area, Hambran, under Mata Kaushalya Devi Pahwa Hospital, Christian Medical College and Hospital, District Ludhiana and under jurisdiction of CHC Sidhwabhet, Punjab, India. 

The study was initiated after appropriate ethical approval (Letter No.: MKD/2021/06/05-1, Dated 05 June 2021). Participants who gave consent after providing detailed description of the present study. Consent was also taken from the parents or guardians where the participants age was less than 18 years. The survey followed ethical standards of the and with the Helsinki Declaration of 1975, as revised in 2013. The anonymity all the study participants were always maintained.

 The study population consisted of permanent family members, both males and females, residing in Hambran village. Participants included in the study were those who were physically and mentally fit and provided verbal consent. Excluded from the study were participants on medications such as chemotherapy drugs, immunosuppressants, long-term nonsteroidal anti-inflammatory drugs (NSAIDs), antiretroviral drugs, bisphosphonates, steroids, or any other medications that could cause oral lesions. Additionally, individuals with a recent history (less than one year) of oral trauma or surgery, those with autoimmune disorders, mental health conditions, uncontrolled diabetes, as well as pregnant and breastfeeding mothers, were not included.

The research was conducted from August 2021 to February 2022, until the desired sample size was achieved. A study conducted in Vidisha, Central India, indicated a prevalence of 8.4% for oral lesions. Based on a 95% confidence interval, the minimum calculated sample size was 119; however, this study included 405 participants.

Oral examinations were performed by trained dental assistants under the supervision of dental specialists. Participants in need of medical or surgical attention were advised to visit the dental outpatient department for further evaluation. Simple random sampling was utilized after creating a list of all household members from the family folders in the field practice area. Available members who met the inclusion criteria were incorporated into the study. An Oral Health Questionnaire developed by the World Health Organization was employed, and the lesions were classified according to the provided guidelines. Data were collected through interviews with the participants.^10^

### Statistical analysis

The data was analysed using MS Excel and IBM SPSS version 20. The results were reported as frequencies and percentages and the association between various variables were assessed using Chi-square test and Multivariate regression model for calculating the Odd’s ratio at 95% confidence interval and p value less than 0.05 was considered as significant. Model fit was checked using Hosmer and Lemeshow test and a p-value of more than 0.05 indicated a good fit. 

## RESULTS

**Figure 1:**
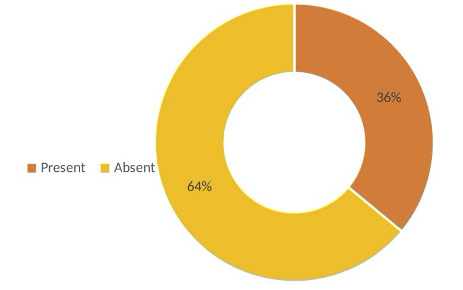
Oral Mucosal Lesions (OML)

A total of 405 participants gave consent to participate in this study, which included 205 (50.6%) males and 200 (49.4%) females. The mean age of the study participants was 44.5 + 16.7 years. The study found that 146 (36%) of the participants were suffering from one or the other oral mucosal lesions (Figure 1). 

Table 1 presents several noteworthy associations between dietary and lifestyle factors and various oral lesions. Among those who consumed fewer fresh fruits, a higher prevalence of candidiasis (30%) was observed. Conversely, individuals with a greater intake of fresh fruits exhibited a more significant association with lesions such as lichen planus (10.5%) and ulcerations, including aphthous ulcers (36.8%), with statistical significance at p<0.05.

**Table 1:** Relation between dietary and tobacco habits with various oral lesions

		Malignant tumour (oral cancer)	Leukoplakia	Lichen planus	Ulceration (aphthous, herpetic, traumatic)	Acute necrotizing ulcerative gingivitis (ANUG)	Candidiasis	Abscess	Other condition	Total	Chi Square p value (P value)
Fresh Fruits	Several times a month	3	3	1	9	1	9	4	0	30	36.25
		10.0%	10.0%	3.3%	30.0%	3.3%	30.0%	13.3%	0.0%	100.0%	(0.001)
	Once a week	11	17	7	31	0	7	24	0	97	
		11.3%	17.5%	7.2%	32.0%	0.0%	7.2%	24.7%	0.0%	100.0%	
	Several times a week	2	1	2	7	1	4	0	2	19	
		10.5%	5.3%	10.5%	36.8%	5.3%	21.1%	0.0%	10.5%	100.0%	
Total		16	21	10	47	2	20	28	2	146	
		11.0%	14.4%	6.8%	32.2%	1.4%	13.7%	19.2%	1.4%	100.0%	
Sweets	Several times a month	2	2	0	10	2	4	5	0	25	31.61
		8.0%	8.0%	0.0%	40.0%	8.0%	16.0%	20.0%	0.0%	100.0%	(0.29)
	Once a week	3	3	1	6	0	3	2	0	18	
		16.7%	16.7%	5.6%	33.3%	0.0%	16.7%	11.1%	0.0%	100.0%	
	Several times a week	2	0	2	6	0	1	1	0	12	
		16.7%	0.0%	16.7%	50.0%	0.0%	8.3%	8.3%	0.0%	100.0%	
	Everyday	9	16	6	21	0	12	16	2	82	
		11.0%	19.5%	7.3%	25.6%	0.0%	14.6%	19.5%	2.4%	100.0%	
	Several times a day	0	0	1	4	0	0	4	0	9	
		0.0%	0.0%	11.1%	44.4%	0.0%	0.0%	44.4%	0.0%	100.0%	
Total		16	21	10	47	2	20	28	2	146	
		11.0%	14.4%	6.8%	32.2%	1.4%	13.7%	19.2%	1.4%	100.0%	
Soft Drinks	Several times a month	11	11	8	33	1	11	23	2	100	19.21
		11.0%	11.0%	8.0%	33.0%	1.0%	11.0%	23.0%	2.0%	100.0%	(0.57)
	Once a week	3	5	1	7	1	5	3	0	25	
		12.0%	20.0%	4.0%	28.0%	4.0%	20.0%	12.0%	0.0%	100.0%	
	Several times a week	2	1	0	4	0	0	1	0	8	
		25.0%	12.5%	0.0%	50.0%	0.0%	0.0%	12.5%	0.0%	100.0%	
	Everyday	0	4	1	3	0	4	1	0	13	
		0.0%	30.8%	7.7%	23.1%	0.0%	30.8%	7.7%	0.0%	100.0%	
Total		16	21	10	47	2	20	28	2	146	
		11.0%	14.4%	6.8%	32.2%	1.4%	13.7%	19.2%	1.4%	100.0%	
Tea/coffee	Less than 1 cup per day	2	12	4	25	2	11	12	0	68	14.17
		2.9%	17.6%	5.9%	36.8%	2.9%	16.2%	17.6%	0.0%	100.0%	(0.04)8
	More than 1 cup per day	14	9	6	22	0	9	16	2	78	
		17.9%	11.5%	7.7%	28.2%	0.0%	11.5%	20.5%	2.6%	100.0%	
Total		16	21	10	47	2	20	28	2	146	
		11.0%	14.4%	6.8%	32.2%	1.4%	13.7%	19.2%	1.4%	100.0%	
Tobacco Smoking	No	12	20	5	36	2	19	17	2	113	17.37
		10.6%	17.7%	4.4%	31.9%	1.8%	16.8%	15.0%	1.8%	100.0%	(0.015)
	Yes	4	1	5	11	0	1	11	0	33	
		12.1%	3.0%	15.2%	33.3%	0.0%	3.0%	33.3%	0.0%	100.0%	
Total		16	21	10	47	2	20	28	2	146	
		11.0%	14.4%	6.8%	32.2%	1.4%	13.7%	19.2%	1.4%	100.0%	
Tobacco Chewing	No	16	19	5	26	1	17	17	2	103	22.53
		15.5%	18.4%	4.9%	25.2%	1.0%	16.5%	16.5%	1.9%	100.0%	(0.002)
	Yes	0	2	5	21	1	3	11	0	43	
		0.0%	4.7%	11.6%	48.8%	2.3%	7.0%	25.6%	0.0%	100.0%	
Total		16	21	10	47	2	20	28	2	146	
		11.0%	14.4%	6.8%	32.2%	1.4%	13.7%	19.2%	1.4%	100.0%	

Daily consumption of sweets was linked to an increased risk of developing ulcerations (44.4%) and abscesses (44.4%). In contrast, the consumption of soft drinks showed associations with leukoplakia (30.8%) and candidiasis (30.8%).

Notably, participants who consumed more than one cup of tea or coffee daily were significantly associated with malignant lesions (17.9%), again at p<0.05. The most concerning findings were the alarming and statistically significant associations (p<0.05) between tobacco smoking and various lesions: malignant lesions (12.2%), lichen planus (15.2%), ulcerations, including aphthous and herpetic ulcers (33.3%), and abscess formations (33.3%). Furthermore, tobacco chewing demonstrated relationships with lichen planus (11.6%), aphthous and herpetic ulcers (48.8%), and abscess formation (26.6%), all of which were statistically significant at p<0.05. 

Table 2 indicates that participants who consumed sweets once a day faced a 4.4-fold increase in the likelihood of having oral lesions. Moreover, those who consumed sweets multiple times a day exhibited a substantially higher risk, with odds 9.2 times greater risk. Daily soft drinks consumers faced 3.6 times higher odds of having these lesions. 

**Table 2:** Association between participants oral status and dietary habits with oral lesions

	B	Wald	p value	OR	95% C.I.	
					Lower	Upper
Pain/Discomfort			
Present				1.0		
Absent	0.0	0.0	0.92	1.0	0.6	1.9
Status of gums/teeth		6.3	0.18			
Very Good				1.0		
Good	1.7	0.8	0.36	5.5	0.1	211.3
Average	1.6	0.7	0.40	4.9	0.1	191.1
Poor	1.0	0.2	0.62	2.6	0.1	111.6
Very Poor	3.5	2.7	0.10	34.7	0.5	2455.3
Cleaning of teeth						
Once a day				1.0		
Twice a day	0.4	0.5	0.47	1.5	0.5	4.6
Fresh Fruits		1.6	0.67			
Several times a month				1.0		
Once a week	-0.1	0.1	0.76	0.9	0.4	2.0
Several times a week	0.6	0.8	0.36	1.8	0.5	6.0
Everyday	-17.4	0.0	1.00	0.0	0.0	
Sweets		16.5	0.00			
Several times a month				1.0		
Once a week	0.1	0.0	0.90	1.1	0.4	2.9
Several times a week	1.0	2.9	0.09	2.8	0.8	9.5
Everyday	1.5	10.0	0.00	4.4	1.8	11.2
Several times a day	2.2	11.3	0.00	9.2	2.5	33.5
Soft Drinks		8.3	0.04			
Several times a month				1.0		
Once a week	-0.2	0.2	0.62	0.8	0.4	1.8
Several times a week	-1.0	2.2	0.14	0.4	0.1	1.4
Everyday	1.3	4.8	0.03	3.6	1.2	11.1
Tea/Coffee						
<1cup/day				1.0		
>1cup/day	0.0	0.0	0.9	1.0	0.5	2.1
Tobacco Smoking						
No				1.0		
Yes	-2.6	48.9	0.0	0.1	0.0	0.2
Tobacco Chewing						
No				1.0		
Yes	-2.1	33.9	0.0	0.1	0.1	0.2

Reference category : No Premalignant Lesion

Model is a good fit: Hosmer and Lemeshow test is Non Significant (p value >0.05)

**Figure 2:**
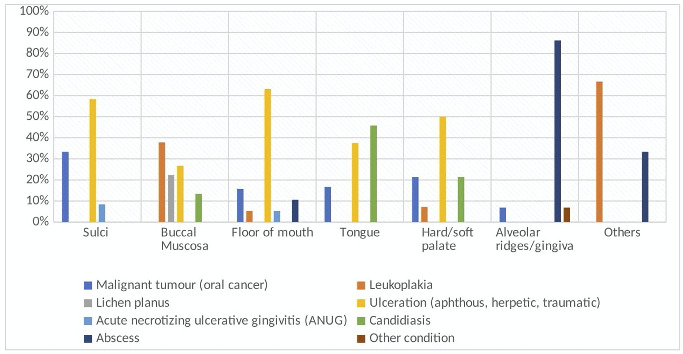
Location of various Oral Mucosal Lesions (OML)

Figure 2 provides valuable insights into the distribution of oral lesions across different areas of the oral cavity. Ulcerations were the predominant lesions, accounting for 58.3% of cases, followed by malignant lesions at 33.3% in the sulci region of oral cavity. Specifically, the buccal mucosa showed a higher association with leukoplakia (37.8%). The floor of the mouth was notably more involved with ulcerations (63.2%) compared to other types of lesions. Candidiasis was most prevalent on the tongue (45.8%), with ulcerations being the second most common lesion (37.5%) in this area. Both the hard and soft palate demonstrated a notable involvement of ulcerations (50.0%), followed by malignant lesions (21.4%) and candidiasis (21.4%). A striking finding was the prevalence of abscesses on alveolar ridges in the study participants, accounting for a substantial 86.2% of cases. Additionally, Acute Necrotizing Ulcerative Gingivitis (ANUG) was observed in both the sulci and the floor of the mouth.

**Figure 3:**
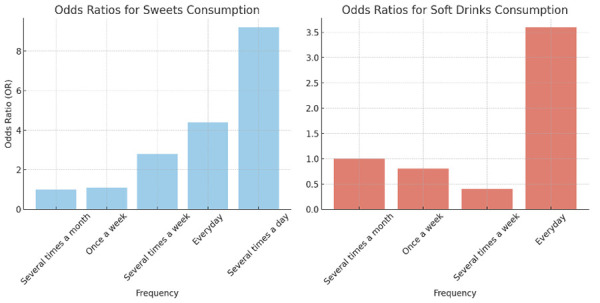
Risk of Oral Mucosal Lesion (OML) with Sweets and Soft Drinks

Figure 3 revealed a significant relationship between sweets consumption and the presence of oral lesions, with odds ratios increasing markedly as the frequency of intake rises. Specifically, individuals consuming sweets "several times a day" exhibit an odds ratio of 9.2, indicating a strong association between frequent sweets consumption and the likelihood of developing oral lesions. Similarly, the consumption of soft drinks also presents a concerning link; those who consume soft drinks "every day" have an odds ratio of 3.6, suggesting a heightened risk of oral lesions compared to individuals who consume soft drinks less frequently. These findings underscore the critical impact of dietary choices on oral health, highlighting the need for public health initiatives focused on reducing high-frequency consumption of sweets and soft drinks to mitigate the risk of oral lesions.

**Figure 4:**
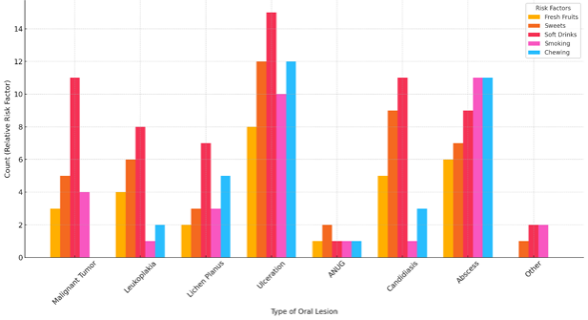
Types of Oral Lesions versus various risk factors

Figure 4 revealed that ulceration is frequently linked to multiple risk factors, while high-frequency sweets consumption correlates strongly with lesions like abscesses and candidiasis. Additionally, both smoking and chewing tobacco are notably associated with conditions like lichen planus and abscesses. This visualization effectively highlights the impact of specific dietary and lifestyle choices on the prevalence of oral lesions, offering insights for targeted prevention strategies. 

## DISCUSSION

The findings of this study provide valuable insights into the associations between dietary habits, lifestyle factors, and the prevalence of oral mucosal lesions (OML) among the study participants. With 36% of participants affected, the relatively high prevalence of OML underscores the significance of oral health awareness and preventive measures in the population.

The study revealed notable associations between dietary habits and the presence of OML. Consumption of fresh fruits appeared to have protective effects against certain lesions such as lichen planus and ulcerations, including aphthous ulcers. These findings are consistent with existing literature highlighting the role of antioxidants and nutrients in maintaining oral health and tissue integrity.^11^^,^^12^ Conversely, frequent consumption of sweets and soft drinks demonstrated positive associations with various OML, emphasizing the detrimental impact of sugar intake on oral tissues and supporting the need for dietary modifications to improve oral health outcomes.^13^^,^^14^ Nutrition Foundation of India recommends that consumption of fruits and vegetables should vary from 150 g/day to 400g/day.^15^ Educating children about food hygiene and dietary practices is essential for promoting lifelong health. Moreover, instilling healthy dietary habits early encourages balanced nutrition, fostering overall well-being and reducing the likelihood of chronic diseases later in life. 

A study conducted by Huang et al^16^ demonstrated that there was an inverse association between Head and Neck Cancer risk and tea consumption, particularly for green tea. Tea drinking appeared to reduce the risk of HNC among regular alcohol drinkers but not among never or occasional alcohol drinkers.^16^ Another study claimed that caffeinated coffee intake was inversely associated with the risk of cancer of the oral cavity and pharynx: the Odds Ratio of 0.96.^17^ The present study showed that more than one cup of tea or coffee daily were significantly associated with malignant lesions (17.9%). This variation can be attributed to excessive consumption of black tea and cooking practices of over boiling of tea. 

Choudhary et al revealed that among the 1,085 subjects with tobacco habits, no lesion was present in 781 (72%) individuals. Tobacco-associated changes in the oral cavity were present in 304 (28%) individuals.^18^ These findings contradict conventional wisdom and merit further investigation into potential confounding factors and underlying mechanisms. The present study demonstrated that tobacco smoking correlated significantly with malignant lesions (12.2%), lichen planus (15.2%), ulcerations (33.3%), and abscess formations (33.3%), while tobacco chewing showed significant associations with lichen planus (11.6%), ulcerations (48.8%), and abscess formation (26.6%). This identifies the risks and stringent implementation of Cigarettes and Other Tobacco Products Act (COTPA) 2003 was enacted in 2004 in India. 

Furthermore, the location-specific distribution of OML highlighted in Figure 2 provides valuable clinical insights. Ulcerations emerged as the predominant lesion type across different oral sites, underscoring the importance of early detection and intervention to prevent complications. The notable prevalence of abscesses on alveolar ridges and the occurrence of Acute Necrotizing Ulcerative Gingivitis (ANUG) further emphasize the diverse spectrum of oral health challenges faced by the study population.

## LIMITATIONS

The present study has a few limitations which includes that the findings may not be generalizable beyond the rural population. Self-reported data on dietary habits may introduce recall and social desirability biases in the results. Environmental and genetic factors were not taken into consideration in this study. Longitudinal studies can be conducted for better accuracy for establishing the causality. 

## CONCLUSION

The findings highlight the need for comprehensive preventive strategies that focus on dietary habits, oral hygiene practices, and lifestyle choices to promote oral health and well-being. Approximately 36% of individuals showed oral mucosal lesions, with diet and lifestyle playing significant roles. Associations were observed between the intake of tea and coffee and malignant lesions, sweets and ulcers, soft drinks, and leukoplakia. Tobacco use also showed notable correlations.

The distribution of oral lesions varied by region, with ulcers being the most common (58.3%), followed by malignancies (33.3%). Leukoplakia was primarily associated with the buccal mucosa, while candidiasis was prevalent on the tongue. Abscesses were notably found on the alveolar ridges, and Acute Necrotizing Ulcerative Gingivitis (ANUG) occurred in the sulci and floor of the mouth.
